# A novel clip-based nerve compression model with high stability and reproducibility for the study of chronic nerve injury associated with neuropathic pain

**DOI:** 10.3389/fnins.2026.1780079

**Published:** 2026-04-07

**Authors:** Zhaoyang Guo, Wentao Zhang, Zhongju Liu, Yuexi Mou, Sai Yu, Hang Zhou, Hang Liu, Haitao Zhou, Xinliang Peng, Zhongyuan He, Rui Deng, Wei Fu, Zhong-liang Deng, Lei Chu

**Affiliations:** 1 Department of Orthopaedics, The Second Affiliated Hospital of Chongqing Medical University, Chongqing, China; 2 Department of Plastic and Maxillofacial Surgery, The Second Affiliated Hospital of Chongqing Medical University, Chongqing, China; 3 Department of Joint Surgery, The Second Affiliated Hospital of Chongqing Medical University, Chongqing, China; 4 Hospital of Chengdu Office of People’s Government of Xizang Autonomous Region, Chengdu, China; 5 Novel Target and Therapeutic Intervention Laboratory, The Second Affiliated Hospital of Chongqing Medical University, Chongqing, China; 6 Department of Pain Management, The Second Affiliated Hospital of Chongqing Medical University, Chongqing, China

**Keywords:** chronic nerve injury, clip-based compression model, neuropathic pain, reproducible animal model, sciatic nerve compression

## Abstract

**Background:**

The pathogenesis of neuropathic pain (NP) is complex and remains incompletely understood, making it essential to establish animal models that can stably and reproducibly recapitulate pain behaviors and functional impairments. The conventional chronic constriction injury (CCI) model of the sciatic nerve is highly operator dependent, resulting in limited reproducibility. This study aimed to establish a NP model with improved stability and reproducibility.

**Methods:**

A NP model was generated by applying sustained and quantifiable mechanical compression to the rat sciatic nerve using a clip device. Pain-related behavioral tests, gait analysis, electrophysiological recordings, and histological and immunofluorescence analyses were conducted to systematically compare and validate the clip-compression model against the CCI model.

**Results:**

Quantitative measurements using a thin-film pressure sensor demonstrated that the clip applied a consistent force of 1.75 ± 0.17 N. Behavioral assessments showed that both the CCI and clip-compression models reliably induced pain hypersensitivity; however, the clip-compression model exhibited a more persistent and stable pain phenotype. Gait and electrophysiological evaluations revealed significant ipsilateral hind-limb gait dysfunction, reduced weight-bearing capacity, and impaired peripheral nerve conduction in the clip-compression model. Histological and immunofluorescence analyses indicated that both models caused nerve fascicle disorganization, myelin damage, and axonal degeneration. Notably, the clip-compression model was associated with relatively milder inflammatory cell infiltration, greater early preservation of myelin architecture, and maintained nerve trunk continuity, more closely resembling the pathological features of chronic nerve compression.

**Conclusion:**

This study establishes and systematically validates a novel clip-based nerve compression model of NP, providing a more stable, reliable, and reproducible animal model for mechanistic studies and therapeutic evaluation in NP research.

## 1 Introduction

Neuropathic pain (NP) is caused by injury or disease affecting the central or peripheral nervous system (Kumagai et al., 2025), with a prevalence of approximately 7%–10% and has become a pressing public health problem (Kuner, 2010; Abboud et al., 2021). NP involves complex alterations in both peripheral and central neural plasticity, and its pathological processes are difficult to investigate systematically and dynamically under clinical conditions (Calvo et al., 2019). Consequently, stable and reproducible animal models have become essential tools for basic research in this field. The development and application of experimental animal models that reliably reproduce pain phenotypes, enable longitudinal tracking of disease progression, and support mechanistic and interventional studies are critical for elucidating the fundamental mechanisms of NP and promoting translational research (Jaggi et al., 2011).

Currently, several animal models of peripheral nerve injury–induced NP have been established, among which the chronic constriction injury (CCI) model is the most widely used (Jaggi et al., 2011; Challa, 2015; Wang et al., 2020). However, this traditional model has notable limitations in practice. The CCI model typically relies on suture ligation, and the degree of constriction and force distribution are highly dependent on operator experience, making standardization and quantitative control difficult and leading to substantial inter-experimental variability. In contrast, nerve crush models often produce injuries that are excessively acute or highly reversible, limiting their ability to stably mimic chronic NP (Jaggi et al., 2011; Challa, 2015; Chen et al., 2020; Wang et al., 2021). Therefore, there is an urgent need for a novel animal model with controllable injury intensity, high reproducibility, and the capacity to reliably induce a chronic pain phenotype.

Based on these considerations, the present study established a new sciatic nerve compression–induced pain model using a clip device. The clamping force of the clip was quantitatively measured, and the successful induction of NP was validated using behavioral assessments and histological analyses. This model provides a more stable and reliable animal model for the study of NP in rats.

## 2 Methods

### 2.1 Mechanical clamping force and opening resistance of the clip-applier system

To quantitatively assess the clamping force applied by the LIGACLIP™ endoscopic rotating applier during clip closure, a thin-film pressure sensor (Runes Kee, China) connected to a signal acquisition system was used. During testing, the sensor was positioned between the two jaws of the applier, with the contact point standardized at one-third of the distance from the clip tip to the hinge, consistent with the predefined clamping position used in modeling. The applier was then closed to the fully clamped state, and the peak sensor output during closure was recorded and converted to clamping force using a calibration curve. A new clip was used for each measurement, and measurements were repeated five times.

To evaluate the opening resistance of the clip, a mini-biomechanical testing device (HRJ Inc., Jinan, China) was used to measure both the compressive force generated during clip formation and the opening force required to separate the clip after formation. One side of the clip was fixed to the testing platform, while compressive or tensile loads were applied to the opposite side at a constant displacement rate. The force–displacement curves were continuously recorded. The maximum compressive force during clip formation and the maximum opening force during clip separation were extracted for analysis. Each clip was tested only once, with five independent repetitions.

### 2.2 Animals

Adult male Sprague–Dawley rats (150 ± 20 g) were purchased from Ensville (Chongqing, China). Animals were housed under controlled temperature and humidity conditions with a 12 h light/12 h dark cycle and had free access to food and water. All experimental procedures complied with the Guidelines for the Care and Use of laboratory animals issued by the chinese ministry of health and were approved by the institutional ethics committee of chongqing medical university (IACUC-SAHCQMU-2023-0056). A randomized, double-blinded, controlled experimental design was applied throughout the study.

### 2.3 Animal model establishment and grouping

**Figure 1:**
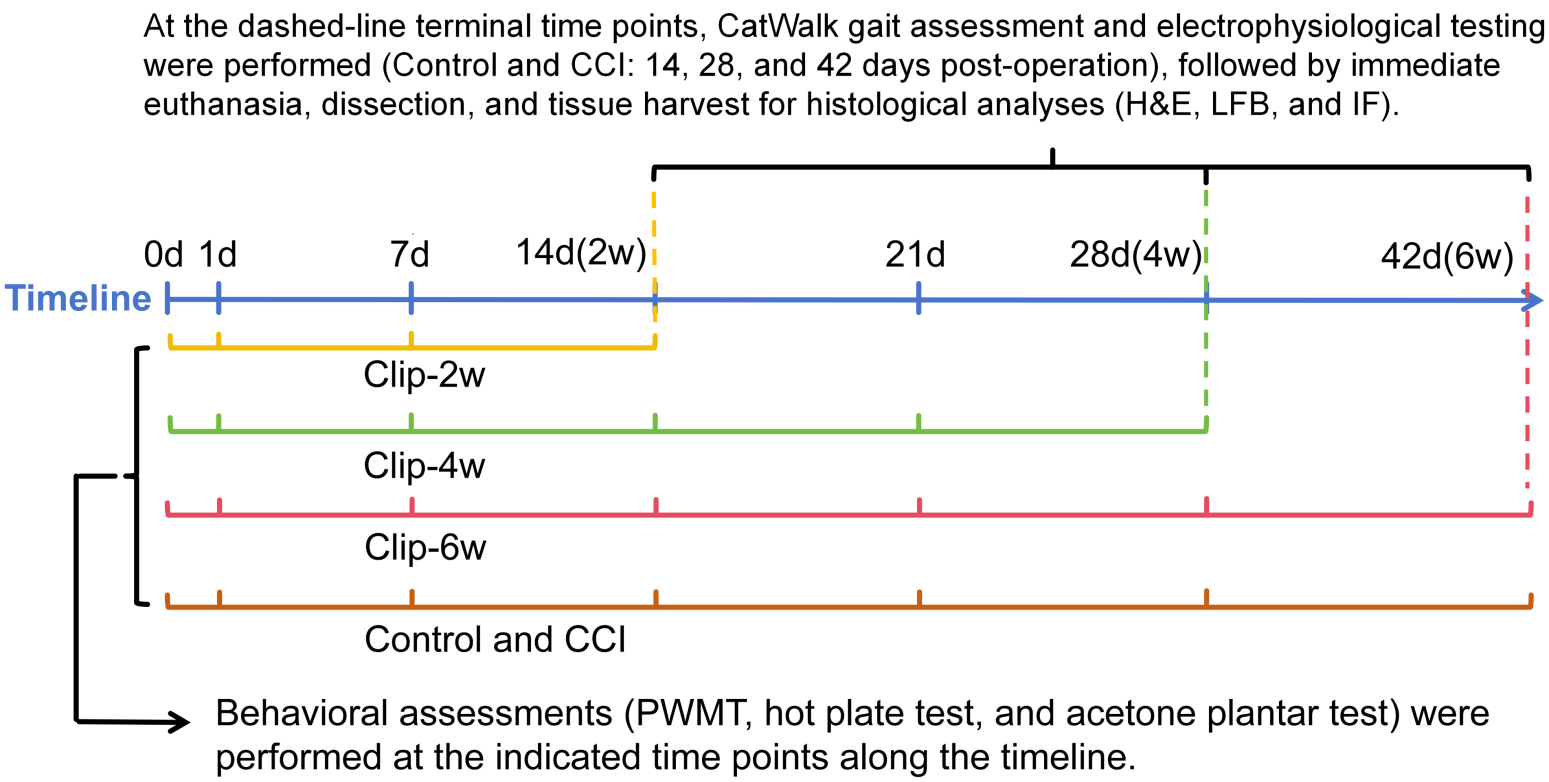
Schematic timeline of experimental assessments and terminal tissue collection for each rat group.

A rat sciatic nerve compression–induced pain model was established using medium-sized clips applied with a LIGACLIP™ endoscopic rotating applier (Ethicon, Ohio, USA). A total of 40 rats were used in this study and were randomly assigned into five groups (*n* = 8 per group): Control, CCI, Clip-2w, Clip-4w, and Clip-6w groups. No animals died, were excluded, or were replaced during the experimental period. The assessment and terminal time points for each group are summarized in Figure 1 and Table 1. After anesthesia with pentobarbital sodium (40 mg/kg), rats were placed in the prone position, and the right hind limb was disinfected to expose the sciatic nerve. We confirm that no auxiliary analgesics (either local anesthetics or systemic analgesics) were administered intraoperatively or postoperatively in this study. In the Control group, the sciatic nerve was exposed and the incision was closed without further manipulation. The CCI model was established according to the method described by Bennett and Xie (1988) In the clip-compression groups, the sciatic nerve was compressed using the LIGACLIP™ applier at a standardized position located approximately 2 cm proximal to the sciatic nerve bifurcation, with the clamping point set at one-third of the distance from the clip tip to the hinge to ensure reproducibility. To minimize inter-animal variability, all surgical procedures were performed by the same investigator. Animals were euthanized by carbon dioxide (CO_2_) asphyxiation at 2, 4, or 6 weeks after surgery, and tissues were collected to evaluate the establishment and stability of chronic NP.

**Table 1:** Experimental schedule of assessments and tissue collection across groups.

Group	Assay	Time point						
		0 d	1 d	7 d	14 d	21 d	28 d	42 d
Control	Behavior tests (PWMT/hot plate/acetone)	✓	✓	✓	✓	✓	✓	✓
	Gait assessment				✓		✓	✓
	Electrophysiological test				✓		✓	✓
	Histology/IF							✓
CCI	Behavior tests (PWMT/hot plate/acetone)	✓	✓	✓	✓	✓	✓	✓
	Gait assessment				✓		✓	✓
	Electrophysiological test				✓		✓	✓
	Histology/IF							✓
Clip-2w	Behavior tests (PWMT/hot plate/acetone)	✓	✓	✓	✓			
	Gait assessment				✓			
	Electrophysiological test				✓			
	Histology/IF				✓			
Clip-4w	Behavior tests (PWMT/hot plate/acetone)	✓	✓	✓	✓	✓	✓	
	Gait assessment						✓	
	Electrophysiological test						✓	
	Histology/IF						✓	
Clip-6w	Behavior tests (PWMT/hot plate/acetone)	✓	✓	✓	✓	✓	✓	✓
	Gait assessment							✓
	Electrophysiological test							✓
	Histology/IF							✓

### 2.4 Behavioral assessments

#### 2.4.1 Paw withdrawal mechanical threshold (PWMT)

Mechanical hypersensitivity was assessed on days 0, 1, 7, 14, 21, 28, and 42 after surgery using von Frey filaments, following the classic up-down method described by Dixon (1980) and Chaplan et al. (1994) to assess mechanical hypersensitivity and estimate the 50% withdrawal threshold. Rats were placed on a metal mesh platform and covered with a transparent acrylic chamber. After a 30-min acclimation period, von Frey filaments (starting at 2 g) were applied perpendicularly to the plantar surface of the hind paw through the mesh until the filament bent slightly into an S shape. Each stimulus lasted 3–5 s with an interval of 10–15 s. Paw withdrawal responses were recorded. Each rat was tested six times with 10-min intervals. The highest and lowest values were excluded, and the remaining values were averaged to obtain the PWMT.

#### 2.4.2 Hot plate test

Thermal nociceptive responses were evaluated on days 0, 1, 7, 14, 21, 28, and 42 after surgery using a hot plate maintained at 50 °C ± 1 °C, following the protocol described by Deuis et al. (2017). The hot plate test was used to assess the nociceptive response of the ipsilateral (injured) hind paw. Each rat was gently placed at the center of the heated plate, and the latency to nocifensive behaviors (jumping, rapid paw withdrawal, repeated paw lifting, or paw licking) was recorded. A cutoff time of 30 s was applied to prevent tissue injury. Each rat was tested six times with 15-min intervals. The highest and lowest values were excluded, and the remaining values were averaged for statistical analysis.

#### 2.4.3 Acetone plantar test

Cold sensitivity was assessed according to the method described by Silva-Cardoso et al. (2021). Rats were placed on a metal mesh platform and allowed to acclimate for 10 min. A total of 100 μL acetone was gently applied to the plantar surface of the right hind paw using an insulin syringe, and behavioral responses were observed for 40 s. Responses were scored as follows: 0, no response; 1, rapid paw withdrawal or brief movement; 2, repeated paw movement; 3, repeated paw movement accompanied by licking. Each rat was tested six times with 15-min intervals. The highest and lowest scores were excluded, and the remaining values were averaged.

#### 2.4.4 Gait analysis

Gait analysis was performed at multiple time points using the CatWalk XT 10.6 system (Noldus). Rats were placed individually at the entrance of the walkway and allowed to traverse the illuminated glass floor voluntarily. Dynamic pressure changes generated fluorescence signals upon paw contact, producing high-contrast footprint images that were recorded by a high-speed camera beneath the walkway. A minimum of three valid runs was collected per rat at each time point. Runs were considered valid only when the animal crossed the walkway continuously without stopping, turning, or obvious hesitation. Runs with excessive speed variation were excluded, and only runs meeting the predefined CatWalk compliance criteria were included in the final analysis. Raw images were semi-automatically processed using CatWalk XT software, including background subtraction and noise filtering, followed by manual identification of footprints (left hind limb, right hind limb, left forelimb, and right forelimb) to ensure accurate classification. Results analysis were performed in a blinded manner. The Sciatic Functional Index (SFI) and Max Contact Max Intensity were calculated for each rat.

### 2.5 Electrophysiological assessment

Electrophysiological recordings were performed using a biological signal acquisition and analysis system (BL-420F, Mengtai, China) with hook electrodes for stimulation and recording of compound muscle action potentials (CMAP). One day before sacrifice, rats were anesthetized and placed in the prone position, and their body temperature was maintained at 37.0 °C ± 0.5 °C using a heating pad throughout the recording procedure. Body temperature stability was monitored using an infrared skin surface temperature probe. The sciatic nerve was exposed, and two bipolar hook-shaped stimulating electrodes were placed on the sciatic nerve with an inter-electrode distance of 1 cm. The active recording electrode was inserted into the belly of the gastrocnemius muscle, the reference electrode was placed in the Achilles tendon region, and the ground electrode was placed at the tail, distant from the stimulation and recording sites, to minimize electrical interference and signal contamination from surrounding muscles. This electrode configuration was standardized for all animals. Before formal recording, a stimulus–response curve was established in each group by gradually increasing the stimulus intensity until the CMAP amplitude reached a plateau. The final stimulation intensity was then set at approximately twofold the intensity required to evoke the maximal CMAP response, ensuring supramaximal stimulation. Rectangular electrical pulses (2.5 V, 0.2 ms duration) were delivered to evoke CMAPs. To improve the reliability of latency-related measurements, the stimulating electrodes were positioned at the same anatomical level of the sciatic nerve in all animals, the hindlimb posture was standardized during recording, and the distance from the stimulating cathode to the recording electrode site was measured using a vernier caliper and kept as constant as possible across animals. The latency (Lm) and amplitude (Am) of the M wave were recorded and analyzed.

### 2.6 Hematoxylin and eosin (H&E) staining

Tissues were fixed in 4% paraformaldehyde for 72 h, followed by graded dehydration and paraffin embedding. Sections with a thickness of 5–8 μm were prepared and stained using a H&E staining kit (Solarbio, China). After deparaffinization in xylene and rehydration through graded ethanol, sections were stained with hematoxylin for 2 min, differentiated in 1% acid alcohol for 3 s, and counterstained with eosin for 45 s. Sections were then dehydrated through graded ethanol, cleared in xylene, and mounted with neutral resin. Images were acquired using a digital pathology slide scanner (KFBIO, China).

### 2.7 Luxol fast blue (LFB) staining

Sciatic nerves were harvested from rats, fixed, embedded, and sectioned as described above. Sections were deparaffinized in xylene, rehydrated through graded ethanol to 95%, and rinsed in distilled water. Sections were then immersed in LFB solution (Solarbio, China) overnight at room temperature. On the following day, sections were rinsed in 95% ethanol and differentiated in lithium carbonate solution (Solarbio, China) for 2–3 s, followed by thorough rinsing in distilled water. Sections were subsequently dehydrated, cleared, and mounted. LFB-stained images were captured using a digital pathology slide scanner (KFBIO, China).

### 2.8 Immunofluorescence (IF) staining

Nerve tissues were harvested, fixed, embedded, sectioned, and rehydrated as described above. Sections were treated with 3% hydrogen peroxide for 15 min, followed by heat-induced antigen retrieval for 20 min. After washing three times with PBS (5 min each), sections were blocked with 5% bovine serum albumin (BSA) for 1 h and incubated overnight at 4 °C with primary antibodies against TUJ1 (1:1000, Abcam, UK) and myelin basic protein (MBP; 1:200, Abcam, UK). On the following day, sections were washed with PBS and incubated with the appropriate fluorescent secondary antibodies for 2 h at room temperature in the dark. Nuclei were counterstained with DAPI (Beyotime, China). Sections were mounted with an anti-fade mounting medium (Beyotime, China) and imaged using a laser confocal microscope (IXplore IX85 Spin, Olympus, Japan). For quantitative analysis, all images were acquired under identical microscope settings. Images were then analyzed using ImageJ software. For each marker, positive signals were separated from background using a fixed threshold applied uniformly to all images within the same staining batch, and the percentage of positive area was calculated. To minimize subjectivity, threshold setting and quantification were performed in a blinded manner using the same analysis criteria across all groups.

### 2.9 Statistical analysis

Statistical analyses were performed using GraphPad Prism version 8.0.2. Comparisons between two groups were conducted using an unpaired, two-tailed Student’s *t*-test. For comparisons involving three or more groups, one-way ANOVA or two-way repeated measures ANOVA was applied, followed by Tukey’s *post hoc* multiple comparisons correction. Results are presented as mean ± SD. *p* < 0.05 was considered statistically significant. All quantitative experiments were repeated in at least three independent experiments.

## 3 Results

### 3.1 Mechanical characterization of the clip-based clamping system

**Figure 2:**
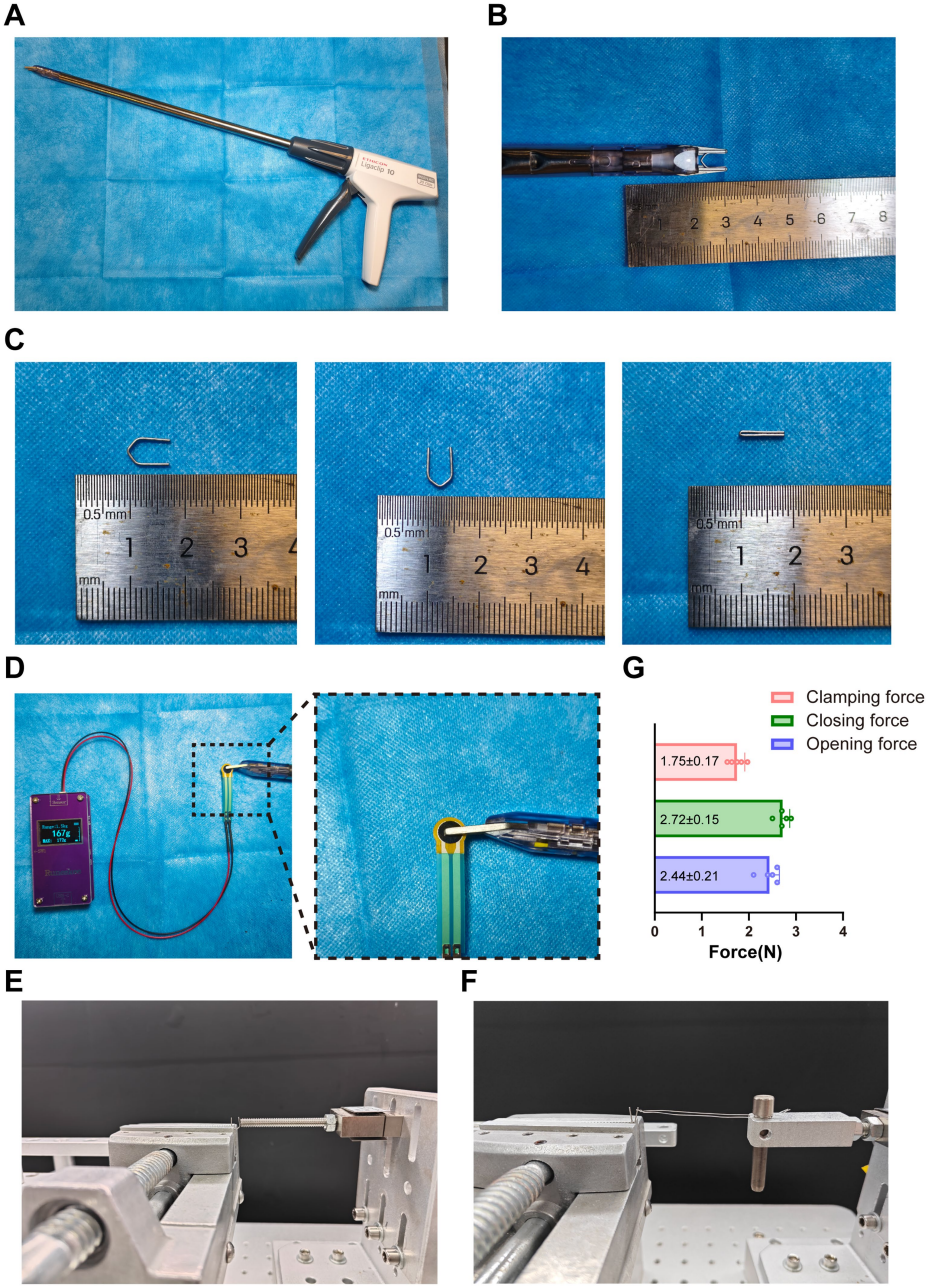
Mechanical characterization of the clip-compression system and clip device. **(A)** Overall appearance of the LIGACLIP™ endoscopic rotating applier used for clip application. **(B)** Close-up view of the distal clamping jaws of the applier. **(C)** Med-size clips used in this study, showing the open configuration and the closed (formed) configuration after clamping, with length and size references. **(D)** Measurement of the clip clamping force using a thin-film pressure sensor, with an enlarged view showing the sensor positioned between the clamping jaws. **(E)** Experimental setup for measuring the compressive/clamping force generated during clip closure (closing force). **(F)** Experimental setup for measuring the deformation force required to reopen the clip after closure (opening force). **(G)** Summary of mechanical force measurements obtained from the closing and opening tests (N); bars represent mean ± SD and dots indicate individual measurements (*n* = 5).

To reduce the large inter-individual variability and non-quantifiable bias inherent to traditional CCI models, a clip-based system was used to establish a standardized sciatic nerve compression load, and its mechanical properties were systematically characterized. As shown in Figures 2A–C, the LIGACLIP™ rotating applier reliably loaded and closed the medium-sized clip, which formed a uniform and consistent closed configuration after deployment.

Mechanical output was first evaluated using a thin-film pressure sensor to record the clamping force during clip closure. The results showed that the clip system generated a clamping force of 1.75 ± 0.17 N (Figure 2D). In addition, a mechanical testing platform was used to measure the deformation-related compressive force generated during clip formation (Figure 2E) and the force required to reopen the clip after formation (Figure 2F). Quantitative analysis revealed a formation compressive force of 2.72 ± 0.15 N and an opening force of 2.44 ± 0.21 N (Figure 2G). These data demonstrate that the clip-based system provides a quantifiable and reproducible compressive load, enabling standardized construction of a sciatic nerve compression injury model.

### 3.2 Clip compression reliably induces NP behaviors

**Figure 3:**
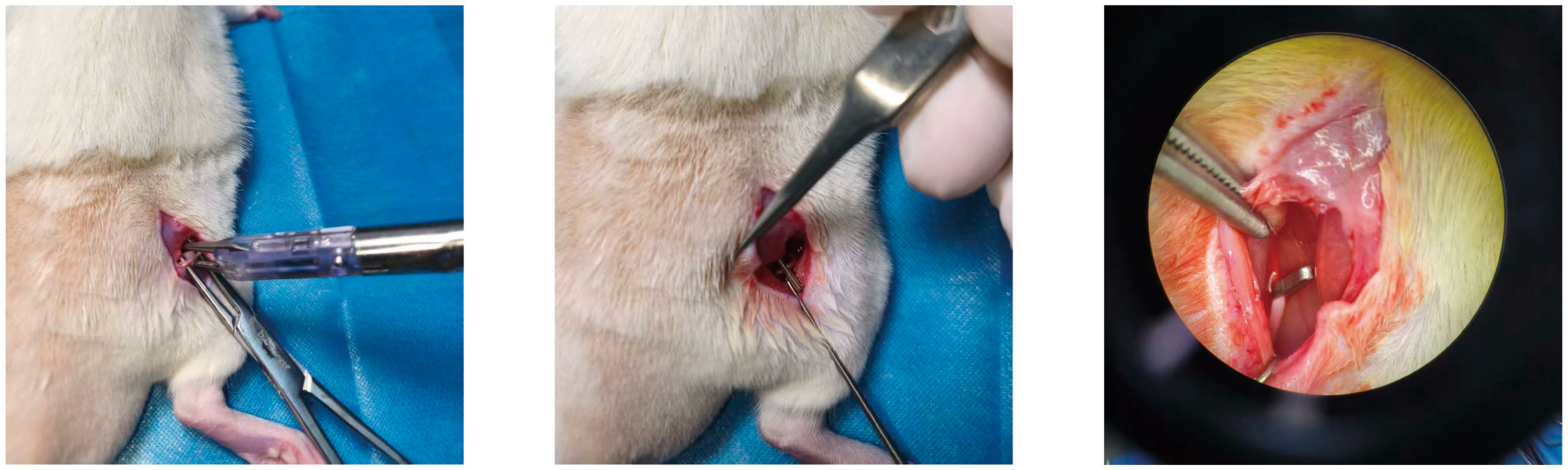
Establishment of the clip-compression–induced NP model in rats.

**Figure 4:**
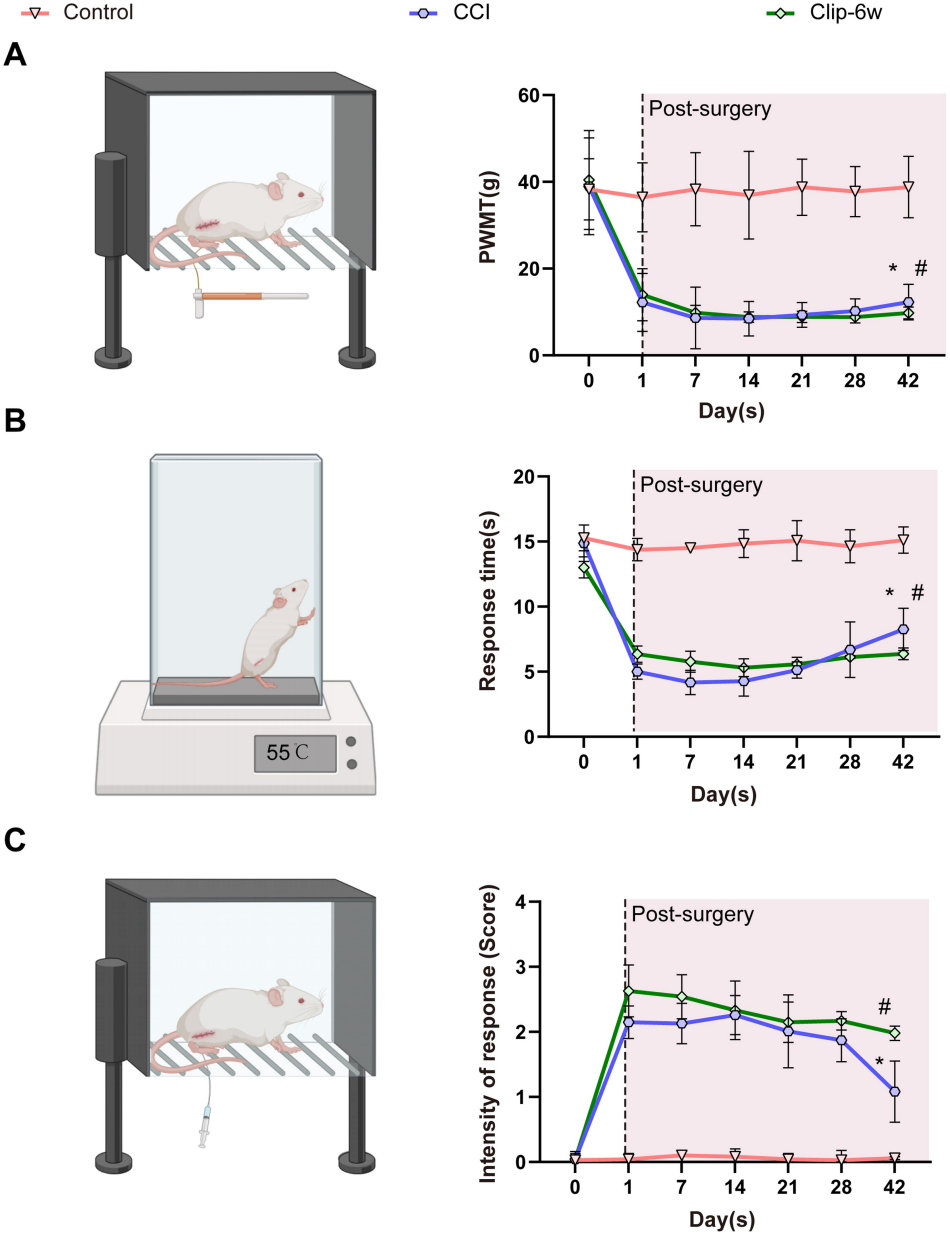
Behavioral assessment of NP in rats subjected to CCI and clip-compression injury. **(A)** Schematic illustration and quantitative analysis of PWMT measured by the von Frey test. **(B)** Schematic illustration and results of the hot plate test showing changes in thermal nociceptive responses. **(C)** Schematic illustration and results of the acetone test for cold sensitivity. PWMT: Paw withdrawal mechanical threshold. *n* = 4, *p < 0.05, comparison between CCI and control groups; #*p* < 0.05, comparison between Clip-6w and control groups.

The PWMT is one of the most used indices for assessing mechanical hypersensitivity in peripheral nerve injury–induced NP. Following model establishment (Figure 3), PWMT was significantly reduced in all experimental groups compared with the Control group, with no significant differences observed among the injury models (Figure 4A).

Thermal nociception was assessed using the hot plate test. From postoperative day 1, the CCI and all clip-compression groups exhibited significantly shortened response latencies compared with the Control group, which remained at low levels from days 7 to 21. At day 28, partial recovery of latency was observed in the CCI and Clip-6w groups. By day 42, the CCI group showed a marked increase in response latency, whereas the Clip-6w group exhibited only a slight, non-significant increase and maintained a relatively low latency (Figure 4B).

Cold hypersensitivity was evaluated using the acetone test. Both the CCI and clip-compression groups showed significantly higher scores than the Control group after surgery. Although a gradual decrease in scores was observed in all experimental groups after day 28, the reduction was most pronounced in the CCI group at day 42. In contrast, the clip-compression groups showed only a modest decline and maintained higher cold sensitivity than the CCI group (Figure 4C).

### 3.3 Clip compression induces sustained gait dysfunction and ipsilateral weight-bearing deficits

**Figure 5:**
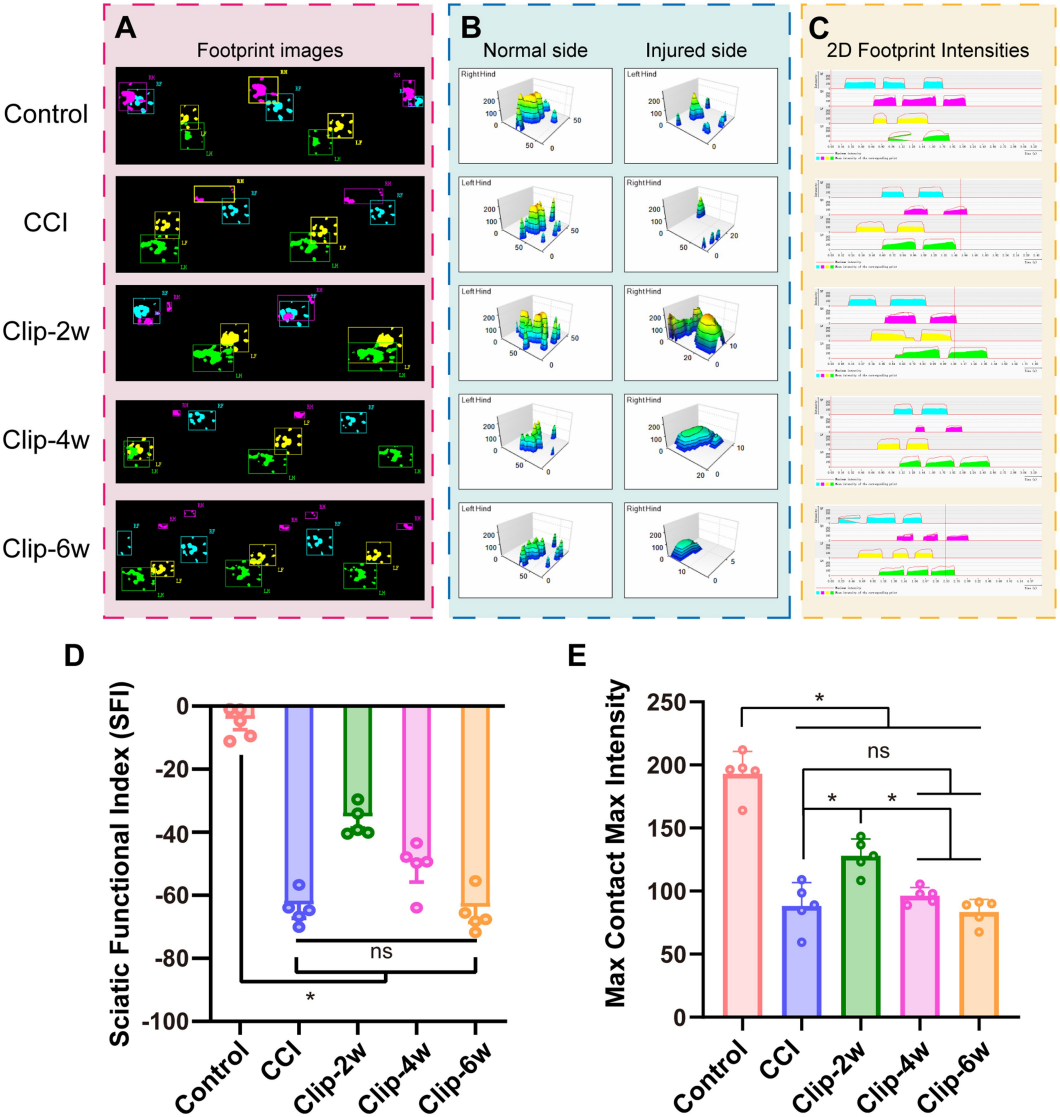
Gait analysis evaluation of rats subjected to CCI and clip-compression. **(A)** Representative footprint images of each group (pink: right hind limb [RH], injured side; green: left hind limb [LH], normal side). **(B)** Representative three-dimensional and **(C)** two-dimensional footprint intensities distribution maps. **(D)** Quantitative analysis of the sciatic functional index. **(E)** Max contact max intensity. *n* = 5, ^*^p < 0.05.

Gait analysis plays a critical role in evaluating motor function recovery and indirectly assessing pain-related behaviors following sciatic nerve injury. In this study, gait was assessed using footprint images, two- and three-dimensional plantar pressure maps, and quantitative parameters including the SFI and maximal contact maximal intensity (MCMI) to comprehensively evaluate gait alterations induced by different injury models (Figures 5A–E).

Compared with the Control group, both the CCI and all clip-compression groups exhibited marked gait impairment in the ipsilateral (right) hind limb. Representative footprint images showed lighter and smaller ipsilateral paw prints with irregular step sequences in injured animals. Consistently, 2D and 3D plantar pressure maps demonstrated reduced peak pressure, decreased contact area, and shortened load duration in the injured hind limb, accompanied by compensatory increases in the contralateral limb.

Quantitative analysis further confirmed these observations. SFI values were significantly reduced in the CCI and all clip-compression groups compared with Controls, indicating substantial motor and gait dysfunction after sciatic nerve injury. No significant differences in SFI were observed between the CCI and clip-compression groups, although the Clip-2w group showed a slightly higher SFI than the other injury groups (Figure 5D). Similarly, MCMI was significantly lower in the CCI and all clip-compression groups than in Controls, reflecting reduced maximal weight-bearing capacity of the injured limb. Among the injury groups, MCMI values were relatively higher in the Clip-2w group and lowest in the Clip-6w group (Figure 5E).

Overall, gait analysis consistently demonstrated that both CCI and clip compression induced sustained ipsilateral unloading and gait dysfunction. Moreover, weight-bearing–related parameters exhibited duration-dependent differences across clip-compression groups, suggesting that the extent of functional impairment is associated with the duration of nerve compression.

### 3.4 Clip compression produces stable and reproducible electrophysiological deficits

**Figure 6:**
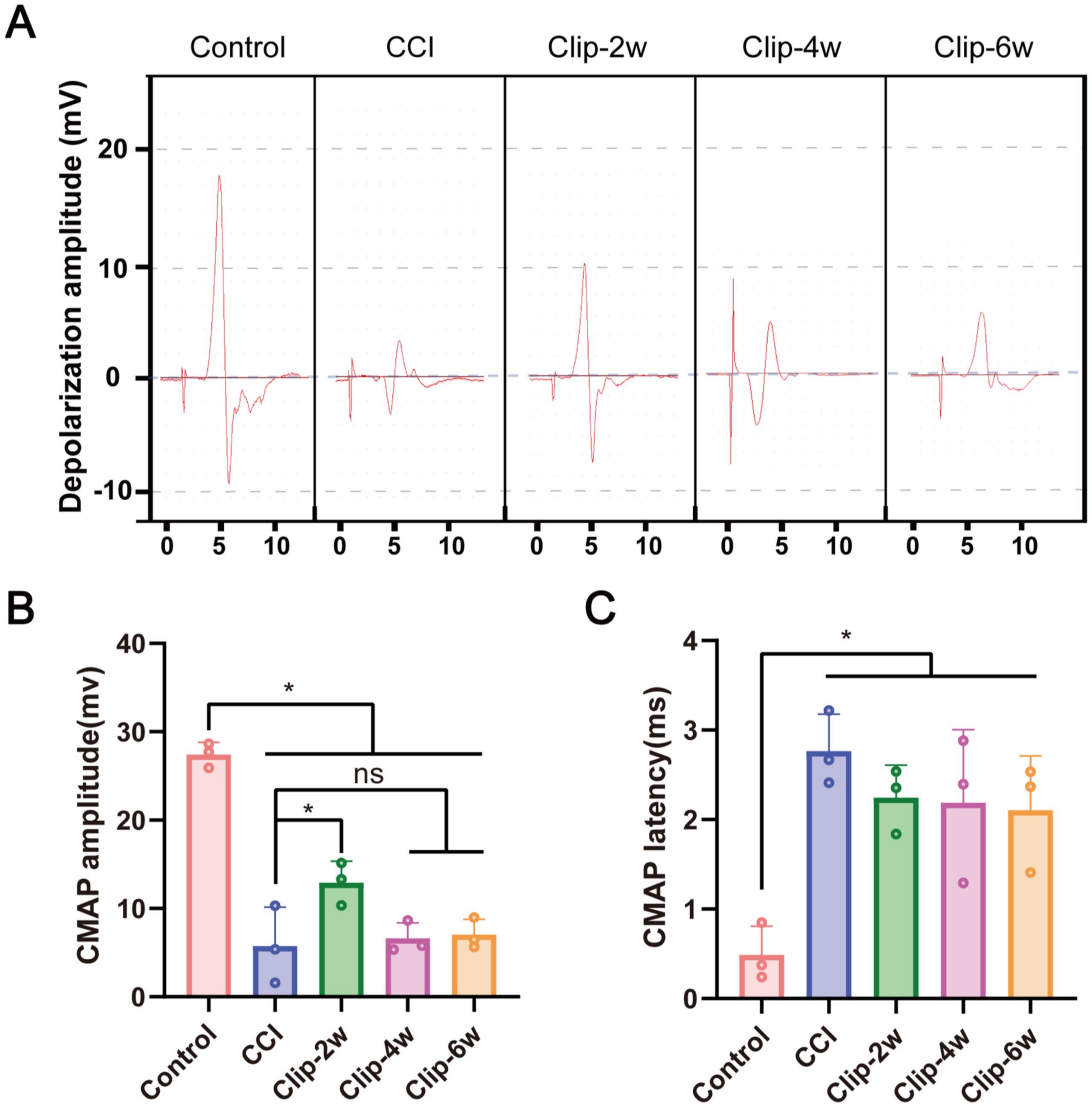
Electrophysiological characteristics in different groups. **(A)** CMAP waveforms in each group. **(B)** CMAP amplitude. **(C)** CMAP latency. CMAP: Compound muscle action potentials. *n* = 3, *p < 0.05.

To objectively evaluate alterations in nerve conduction following sciatic nerve injury, CMAP was recorded in all groups. In the Control group, CMAP recordings exhibited high amplitudes, well-defined waveforms, and good reproducibility. In contrast, the CCI and all clip-compression groups showed markedly reduced CMAP amplitudes (Figure 6A).

Quantitative analysis revealed that CMAP amplitudes were significantly decreased in the CCI group compared with Controls. Within the clip-compression model, the Clip-2w group displayed slightly higher CMAP amplitudes than the other injury groups (*p* = 0.042), whereas the Clip-4w and Clip-6w groups maintained low amplitudes comparable to those observed in the CCI group (Figure 6B). Notably, the clip-compression groups exhibited substantially lower within-group variability, as reflected by smaller standard deviations and reduced inter-individual dispersion, resulting in a narrower range of CMAP values compared with the CCI group.

Analysis of CMAP latency further demonstrated a significant prolongation in both the CCI and clip-compression groups relative to Controls, with no significant differences observed between the two injury models (Figure 6C). These findings indicate that the clip-compression model effectively reproduces sciatic nerve conduction deficits while exhibiting improved intra-group consistency compared with the traditional CCI model.

### 3.5 CCI and clip compression induce sciatic nerve structural damage accompanied by myelin abnormalities

**Figure 7:**
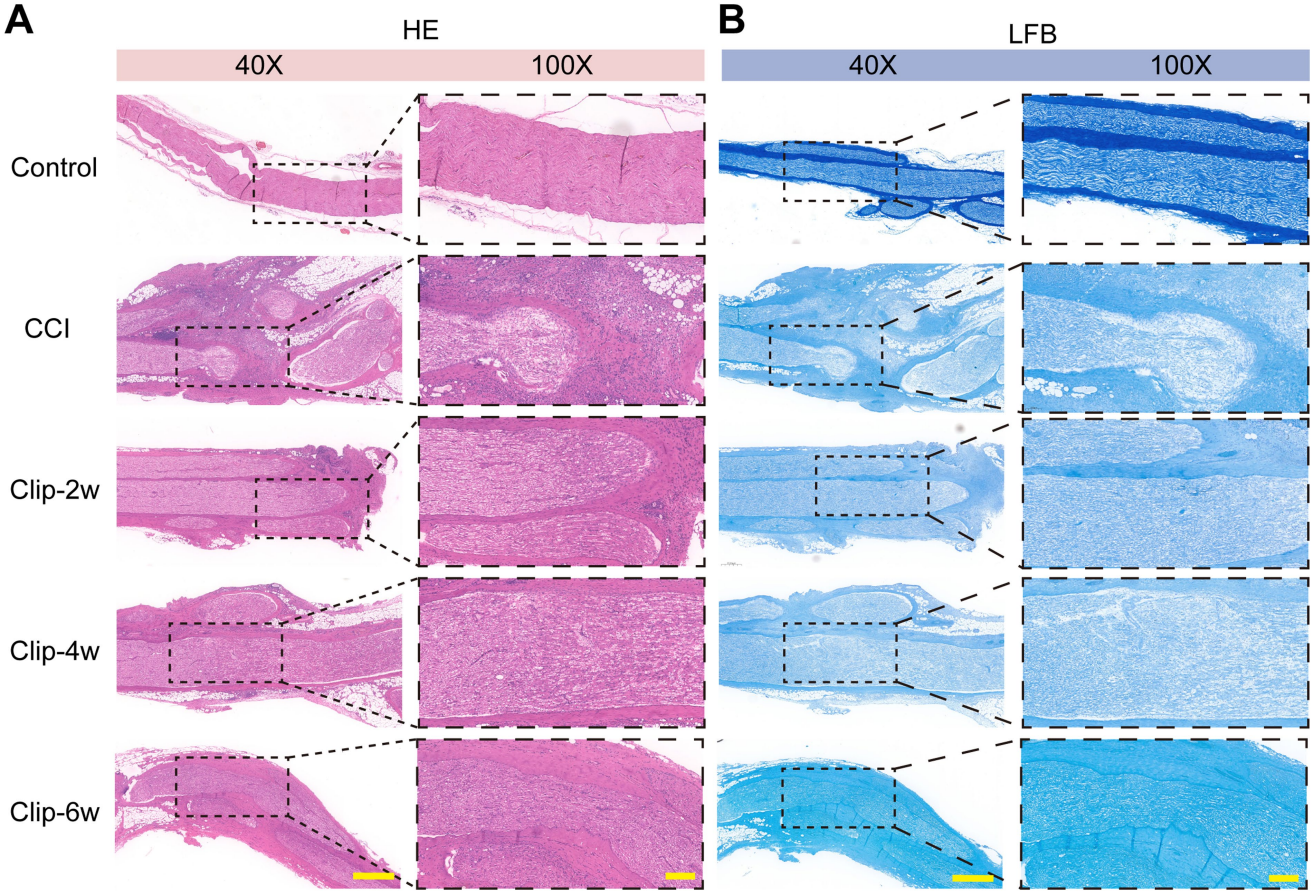
Histopathological staining of sciatic nerves in rats subjected to CCI and clip-compression models. **(A)** H&E staining of sciatic nerves in each group (bar: 40×, 300 μm; 100×, 100 μm). **(B)** LFB staining of sciatic nerves in each group (bar: 40×, 300 μm; 100×, 100 μm). H&E: Hematoxylin and eosin; LFB: Luxol fast blue.

H&E staining revealed that, compared with the Control group, the CCI group exhibited pronounced structural disorganization at the injury site, characterized by blurred nerve fascicle boundaries, loosened intrafascicular architecture, and typical degenerative changes, including disruption of myelin-like structures and formation of myelin digestion chambers associated with Wallerian degeneration. Extensive infiltration of inflammatory cells, such as lymphocytes, was observed in the CCI group, indicating a robust local inflammatory response. Notably, discontinuity of nerve fascicles and widening of perineural spaces with edema-like changes were evident, suggesting more severe perineural structural damage and fatty infiltration in the CCI model (Figure 7A).

Structural abnormalities were also observed in the clip-compression groups. The Clip-2w group primarily showed mild nerve fascicle enlargement and edema, relatively loose intrafascicular organization, and occasional myelin digestion chambers. In contrast, the Clip-4w and Clip-6w groups exhibited more pronounced morphological alterations, including aggravated fascicular remodeling, increased myelin digestion chambers, and reduced myelin-like structures. Overall, however, inflammatory cell infiltration in the clip-compression groups was relatively milder than that observed in the CCI group (Figure 7A).

LFB staining further confirmed these injury-related changes. In the Control group, myelin staining was continuous and uniformly dark blue, indicating intact myelin structure. In contrast, the CCI group showed markedly reduced staining intensity with disrupted continuity, reflecting severe myelin damage. All clip-compression groups also exhibited attenuated and heterogeneous myelin staining, consistent with a reduction in myelin content (Figure 7B).

**Figure 8:**
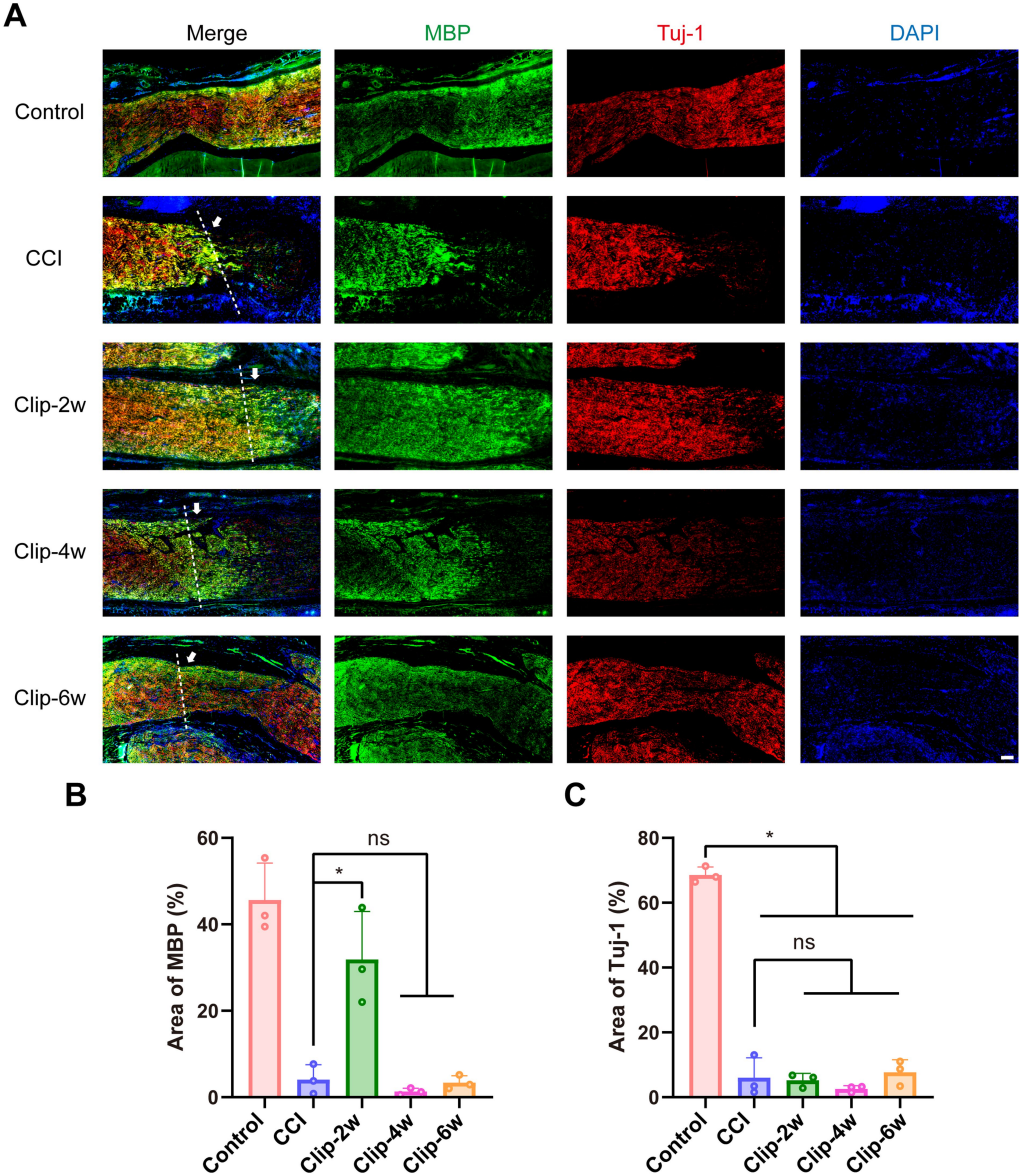
IF staining of sciatic nerves in rats subjected to CCI and clip-compression models. **(A)** IF staining of MBP and Tuj-1 in sciatic nerves from CCI and clip-compression model rats (white arrows and white dashed lines indicate the surgical sites) (bar: 200 μm). **(B)** Percentage of MBP-positive area. **(C)** Percentage of Tuj-1-positive area. *n* = 3, *p < 0.05.

To further assess changes in myelin and axonal structures after sciatic nerve injury, IF staining was performed using TUJ1 (neuronal marker, red) and myelin basic protein (MBP; green). Compared with Controls, the CCI group showed markedly reduced and fragmented MBP and TUJ1 immunoreactivity at the injury site, accompanied by extensive inflammatory cell accumulation in the surrounding tissue, indicating substantial disruption of myelin, axons, and nerve fibers (Figure 8A). In the clip-compression groups, attenuation of MBP and TUJ1 signals was also observed. The Clip-2w group retained relatively higher MBP positive area than the CCI group, whereas the Clip-4w and Clip-6w groups showed a pronounced reduction and more fragmented distribution of MBP-positive areas, comparable to the CCI group (Figure 8B). Quantitative analysis further demonstrated that TUJ1-positive areas were significantly reduced in all injury groups compared with Controls, while no statistically significant differences were detected between the CCI and clip-compression groups (Figure 8C).

**Figure 9:**
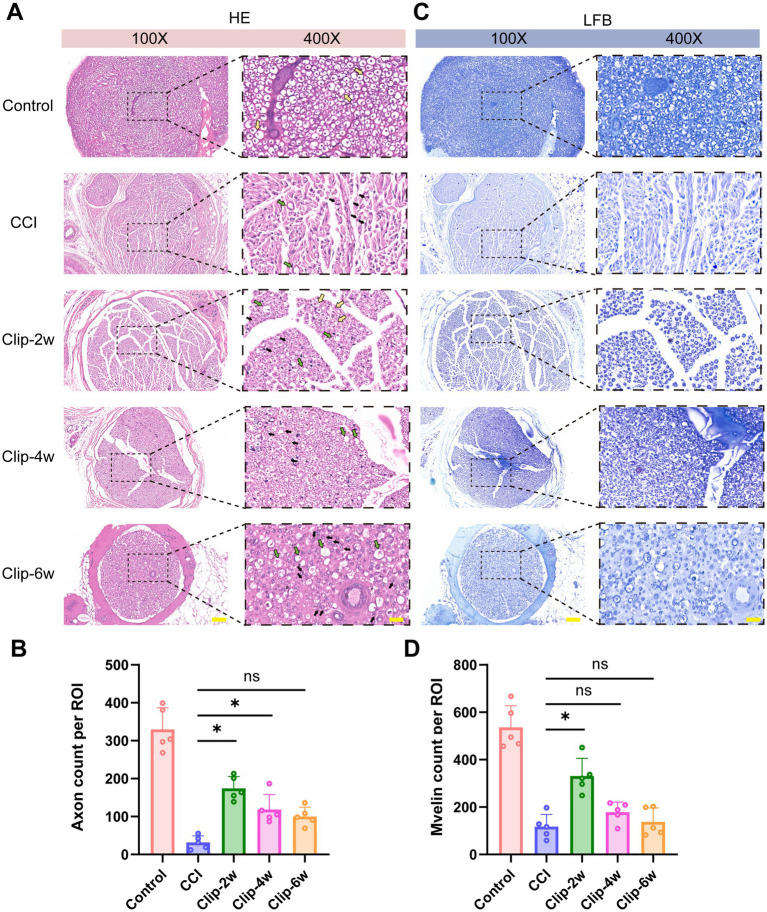
Histological staining of sciatic nerve transverse sections in each group. **(A)** Representative H&E staining images of sciatic nerve transverse sections in each group (bars: 100×, 100 μm; 400×, 25 μm). **(B)** Semi-quantitative analysis of axon counts. **(C)** Representative LFB staining images of sciatic nerve transverse sections in each group (bars: 100×, 100 μm; 400×, 25 μm). **(D)** Semi-quantitative analysis of myelin sheath counts. Images were acquired at 400 × magnification and analyzed using a standardized rectangular region of interest (ROI: 300 × 200 μm). Yellow arrows indicate normal myelinated nerve fiber structures. Green arrows indicate axonal loss with myelin sheath edema. Black arrows indicate demyelinating changes. *n* = 5, *p < 0.05. ns, No statistical significance.

Further evaluation of sciatic nerve transverse sections by H&E staining, as shown in Figure 9, revealed that the Control group exhibited intact and compact nerve architecture with uniformly distributed normal myelinated nerve fibers, in which the boundaries of axons and myelin sheaths were clearly distinguishable (yellow arrows). In contrast, the CCI group showed marked disruption of transverse nerve structure, characterized by a substantial reduction in intact myelinated nerve fibers, accompanied by extensive axonal loss (green arrows), myelin sheath edema, and demyelinating changes (black arrows), indicating severe nerve degeneration (Figure 9A).

The clip-compression groups also exhibited pathological alterations of varying severity. In the Clip-2w group, some relatively intact myelinated nerve fibers were still preserved, although focal axonal loss and myelin abnormalities were already evident. With prolonged compression, the Clip-4w and Clip-6w groups showed a further reduction in normal myelinated nerve fibers, together with more pronounced axonal loss, myelin swelling, and demyelination, suggesting progressively aggravated structural damage. Semi-quantitative analysis further supported these histological findings. Compared with the Control group, axon counts were significantly reduced in all injury groups (Figure 9B), with the greatest decrease observed in the CCI group, whereas the Clip-2w group retained relatively higher axon counts than the Clip-4w and Clip-6w groups. Similarly, myelin sheath counts were also markedly decreased in all injury groups (Figure 9D), with the most pronounced reduction in the CCI group, while the Clip-2w group preserved more myelin structures than the longer-duration compression groups. In addition, semi-quantitative analysis of inflammatory cell infiltration showed that inflammatory cell counts were markedly increased in the CCI group compared with the Control group (Figure 9E), consistent with the prominent inflammatory response observed in the H&E staining. The clip-compression groups also exhibited varying degrees of inflammatory cell infiltration, although the overall level was lower than that in the CCI group. Collectively, these findings indicate that both CCI and clip compression induce substantial structural damage and inflammatory responses in the sciatic nerve, whereas clip compression causes milder injury and inflammation than CCI.

## 4 Discussion

The key mechanisms underlying NP remain incompletely understood, and the establishment of animal models that can stably and reproducibly recapitulate pain behaviors and functional impairments is essential for elucidating disease mechanisms and evaluating therapeutic strategies. Since its introduction by Bennett and Xie, the CCI model has become a classic and widely used model of NP, designed to mimic pain hypersensitivity resulting from clinically relevant chronic compressive nerve injury (Zhou et al., 2025). However, the CCI model has notable limitations. The severity of injury is difficult to precisely control, and even minor differences in ligation tightness among experimenters can markedly influence the number and type of nerve fibers affected. Previous studies have shown that variations in suture tension lead to poor reproducibility of experimental outcomes, rendering the CCI model highly dependent on operator experience and technical skill. Consequently, despite its broad use and clinical relevance, the limited reproducibility, poor controllability of injury severity, and strong operator dependence restrict the utility of the CCI model in studies requiring precise and standardized assessment of nerve function.

To address these limitations, the present study established and systematically evaluated a clip-based model of sustained sciatic nerve compression to simulate the development and progression of NP following peripheral nerve injury. Previous work by Chen et al. (2020) demonstrated that introducing controllable tensile forces into the CCI paradigm can generate nerve injury models with graded severity and improved reproducibility. Compared with traditional loose ligation, clip-based compression minimizes operator-dependent variability and achieves more consistent injury levels across experiments. Although such compression models offer clear advantages in terms of standardization and reproducibility, whether they can induce NP–related behavioral phenotypes comparable to or more robust than those produced by the classic CCI model requires experimental validation. Therefore, in the present study, we performed a systematic comparison of pain-related behavioral outcomes between the clip-compression and CCI models.

Mechanical allodynia is a key sensory feature of NP and is clinically referred to as tactile allodynia (de-la-Llave-Rincón et al., 2012). In the present study, both the CCI and all clip-compression groups exhibited a significant and sustained reduction in PWMT from the early postoperative period, consistent with previous observations in NP models and indicating that both suture ligation and clip-compression can reliably induce a typical mechanical allodynia phenotype (Zhang et al., 2014; Cui et al., 2023; Guo et al., 2023). Thermal and cold sensitivity, which are also commonly assessed in NP models, were similarly increased in both the CCI and clip-compression groups, however, differences emerged over longer time scales. Specifically, pain hypersensitivity in the CCI model showed partial attenuation after 28 days, whereas the clip-compression model maintained a longer-lasting and more stable pain phenotype (Cohen and Mao, 2014). These behavioral findings indicate that clip-based compression effectively induces nociceptive responses and suggest a potential advantage in sustaining pain-related behaviors over time.

Although behavioral assessments demonstrated that both the CCI and clip-compression models reliably induced mechanical, thermal, and cold hypersensitivity, clinical NP is typically accompanied not only by sensory abnormalities but also by motor dysfunction, reduced weight-bearing capacity, and impaired nerve conduction (Hancher, 2001; Nashed et al., 2010; Doneddu et al., 2023). Accordingly, functional gait analysis revealed that both the CCI and clip-compression models produced sustained gait impairment in the ipsilateral hind limb, consistent with previous studies on functional recovery following peripheral nerve injury (Yu et al., 2001; Finnerup et al., 2021). Notably, the clip-compression model exhibited duration-dependent differences in weight-bearing–related parameters. Electrophysiological assessments further confirmed the effectiveness of the clip-compression model in reproducing functional deficits associated with peripheral nerve injury.

Histological evidence provided a clear structural basis for the behavioral and functional alterations observed in this study. H&E and LFB staining demonstrated that both the CCI and clip-compression models induced nerve fascicle disorganization, intrafascicular loosening, myelin damage, and the formation of myelin digestion chambers (Lu et al., 2025) associated with Wallerian degeneration. These morphological features are characteristic of peripheral nerve injury (Shiokawa et al., 1988; Dubový, 2011; Tricaud and Park, 2017). As described by Rotshenker (2011), innate immune responses during Wallerian degeneration generate chemokines and inflammatory mediators that promote myelin fragmentation and clearance, contribute to peripheral nerve sensitization, and enhance pain sensitivity; mechanical and thermal hypersensitivity may, in part, be associated with the upregulation of these mediators. Consistent with these observations, IF analysis in our study showed markedly reduced and fragmented MBP and TUJ1 immunoreactivity in the injured regions of both the CCI and clip-compression groups, indicating substantial disruption of myelin and axonal structures. Notably, the Clip-2w group retained a relatively larger MBP-positive area, suggesting that nerve injury in the clip-compression model progresses in a more gradual manner rather than as an acute destructive insult. This progressive pattern resembles that observed in certain chronic compression injury models, in which myelin degeneration occurs more slowly and macrophage responses are typically delayed and not accompanied by extensive hematogenous inflammatory infiltration. It should be noted that no significant differences in TUJ1-positive area were detected between the CCI and clip-compression groups, which may reflect the temporal dynamics of axonal degeneration (Wei et al., 2019). Previous studies have suggested that axonal degeneration often precedes myelin breakdown and may not be fully captured by immunolabeling at a single time point (Dubový, 2011). Therefore, more detailed temporal analyses may be required to further elucidate the dynamic relationship between axonal injury and pain-related behaviors.

In addition, we observed that the traditional CCI model was associated with extensive inflammatory cell infiltration at the injury site, accompanied by disruption of tissue continuity and widening of interstitial spaces with edema-like changes. These findings indicate that CCI induces not only intrafascicular structural damage but also pronounced disturbances in the perineural microenvironment, whereas the inflammatory response in the clip-compression model was relatively mild. This difference may be related to the superior biocompatibility of the titanium material used in the clip (Toita et al., 2022). Previous studies have shown that local inflammatory reactions induced by titanium clips are rare and that delayed foreign-body responses are observed only in exceptional cases, largely attributed to the spontaneously formed titanium oxide protective layer that reduces immunogenic stimulation (Ho and Ashour, 2010; Tatezawa et al., 2023). Similarly, titanium staples used clinically for gastrointestinal anastomosis have been reported to elicit minimal tissue reactions (Lim et al., 2008), further supporting the notion that titanium itself is unlikely to provoke a strong immune response. Moreover, clip- compression injury was mainly limited to myelin damage and axonal interruption, while the continuity of the nerve trunk and surrounding connective tissue was preserved. This preservation suggests that proximal and distal nerve segments remain anatomically connected, which may facilitate axonal regeneration along original pathways in the distal Wallerian degeneration environment (Zhou et al., 2025). Such a partially injurious yet potentially reversible injury pattern more closely resembles the pathological features of chronic compressive neuropathies observed clinically and is conducive to the investigation of long-term neuropathic changes (Furlanetto et al., 2025).

Several limitations of this study should be acknowledged. Although the mechanical output of the clip-compression procedure was quantitatively characterized, the actual local stress distribution experienced by the nerve may still be influenced by factors such as nerve diameter, tissue thickness, and the extent of surrounding tissue coverage. Future studies incorporating more refined biomechanical measurements or higher-resolution morphological analyses may further strengthen the quantitative assessment. Second, although inflammatory cell infiltration was evaluated by histological examination and semi-quantitative analysis of H&E-stained sciatic nerve transverse sections, this approach lacks cell-type specificity. In addition, the present work primarily focused on peripheral structural and phenotypic validation; mechanisms involving central sensitization, neuroimmune interactions, and dynamic changes in specific cellular subpopulations warrant further investigation.

## 5 Conclusion

This study establishes and systematically validates a novel clip-based sciatic nerve compression model of NP. By quantifying compressive force and minimizing operator-dependent variability, this model demonstrates stable and reproducible NP–related phenotypes across behavioral, gait, histological, and IF analyses. The clip-compression model thus provides a more reliable and reproducible animal model for mechanistic studies and therapeutic evaluation in NP research.

## Data Availability

The raw data supporting the conclusions of this article will be made available by the authors, without undue reservation.
